# How much might a society spend on life-saving interventions at different ages while remaining cost-effective? A case study in a country with detailed data

**DOI:** 10.1186/s12963-015-0052-2

**Published:** 2015-07-08

**Authors:** Giorgi Kvizhinadze, Nick Wilson, Nisha Nair, Melissa McLeod, Tony Blakely

**Affiliations:** Department of Public Health, University of Otago, Wellington, PO Box 7343, Wellington New Zealand

**Keywords:** Health-adjusted life-years (HALYs), Health-adjusted life expectancy (HALE), Cost-effective, Cost-effectiveness threshold, Age, Quality-adjusted life-years (QALYs), Morbidity, Health system costs

## Abstract

**Objective:**

We aimed to estimate the maximum intervention cost (EMIC) a society could invest in a life-saving intervention at different ages while remaining cost-effective according to a user-specified cost-effectiveness threshold.

**Methods:**

New Zealand (NZ) was used as a case study, and a health system perspective was taken. Data from NZ life tables and morbidity data from a burden of disease study were used to estimate health-adjusted life-years (HALYs) gained by a life-saving intervention. Health system costs were estimated from a national database of all publicly funded health events (hospitalizations, outpatient events, pharmaceuticals, etc.). For illustrative purposes we followed the WHO-CHOICE approach and used a cost-effectiveness threshold of the gross domestic product (GDP) per capita (NZ$45,000 or US$30,000 per HALY). We then calculated EMICs for an “ideal” life-saving intervention that fully returned survivors to the same average morbidity, mortality, and cost trajectories as the rest of their cohort.

**Findings:**

The EMIC of the “ideal” life-saving intervention varied markedly by age: NZ$1.3 million (US$880,000) for an intervention to save the life of a child, NZ$0.8 million (US$540,000) for a 50-year-old, and NZ$0.235 million (US$158,000) for an 80-year-old. These results were predictably very sensitive to the choice of discount rate and to the selected cost-effectiveness threshold. Using WHO data, we produced an online calculator to allow the performance of similar calculations for all other countries.

**Conclusions:**

We present an approach to estimating maximal cost-effective investment in life-saving health interventions, under various assumptions. Our online calculator allows this approach to be applied in other countries. Policymakers could use these estimates as a rapid screening tool to determine if more detailed cost-effectiveness analyses of potential life-saving interventions might be worthwhile or which proposed life-saving interventions are very unlikely to benefit from such additional research.

**Electronic supplementary material:**

The online version of this article (doi:10.1186/s12963-015-0052-2) contains supplementary material, which is available to authorized users.

## Introduction

Many countries spend a substantial proportion of their gross domestic product (GDP) on health, e.g., at least 10 % in high-income countries such as New Zealand [1, 2]. The rapid rise in health spending at a rate faster than GDP growth (due to income growth, technological change, population growth, and aging), is unlikely to be tenable in the long term [3–5]. Hence policymakers in health, as in other sectors, need to take increasing care in allocating finite health resources in ways that maximize health benefits for the population.

In the ideal world policymakers would probably have the results of studies that identified the country-specific willingness-to-pay value that society places on saving a life at different ages. In such an ideal world, they may also have a methodologically compatible league table of life-saving and health-gaining interventions – to inform the “next best” intervention to adopt from a cost-effectiveness perspective within a government’s available health budget. But most countries are a long way from having such information available, and indeed they often have limited resources for conducting new cost-effectiveness analyses (which can take many months and cost tens of thousands of dollars per intervention modeled).

Given these problems we aimed to provide information to inform policymakers so that they could more readily determine if more detailed cost-effectiveness analyses of potential life-saving interventions might be worthwhile or which proposed interventions are very unlikely to benefit from such additional research. We just focus here on the issue of cost-effectiveness but note that this is just one component of appropriate decision-making and that many other components [6–12] might reasonably be weighted as also being important, e.g., equity impacts, public acceptability, feasibility, and impact on non-health co-benefits (e.g., greenhouse gas reductions).

We selected New Zealand as a case study country given that it has recently completed a national burden of disease study [13] and has recently produced detailed estimates of health system costs by age group [14]. Furthermore, it is like most countries in having no official threshold for determining what is cost-effective in the health sector and so has to rely on crude measures such as the GDP per capita expenditure to gain a QALY (as used in recent New Zealand-based studies, e.g., [15, 16]). It is also a country where cost-effectiveness considerations might sometimes appropriately be outweighed by concerns for achieving health equity, given the need to improve health for the Māori (indigenous) population [17].

## Methods

Our specific aim in this study was to estimate the maximum amount society could invest up front in a hypothetical life-saving health intervention at different ages while remaining cost-effective. For the rest of this paper, we term this upfront amount “estimated maximum intervention cost,” or EMIC. For the “ideal intervention” EMIC, the death prevented is not one from a pre-existing chronic disease that shortens life expectancy. Rather, it is death from a relatively acute, “short and sharp” disease or injury, where the treatment saves the person’s life and then returns them to expected health, having the same average morbidity and mortality as the average citizen of their age and sex. This approach can be used to estimate the maximum amount society should spend (for different ages) on treatment interventions for acute conditions such as life-threatening infectious diseases or highly treatable cancers of relatively short duration such as testicular cancer. Such an analysis requires data on the health-adjusted life expectancy of the average citizen and the future stream of health spending that they will consume over their lifetime.

A cost-effectiveness threshold (CET) is the amount of money that society is willing to pay for an additional unit of health gain, usually one quality-adjusted life-year (QALY) gained [18], or one disability-adjusted life-year (DALY) averted. We note, however, that there is no universal agreement on estimates of a cost-effectiveness threshold [19–22], how these estimates should be derived [23, 24], or even whether a fixed threshold should be kept at all [25]. For illustrative purposes, we use the WHO-CHOICE guidance of GDP per capita as a starting CET but provide an online calculator that allows the user to specify other CETs. In this paper we use the generic term HALY (health-adjusted life-years) to cover both QALYs and DALYs. If the cost of the health intervention to gain one HALY is less than the CET, then the intervention is deemed cost-effective.

The estimated maximum intervention cost or EMIC for a given age k was calculated using the following formula:

$$ EMIC=A(max)=\left(CET \times HALY(k)\right) - C(k) $$

where A (max) is the theoretical maximum of one-off direct cost of the life-saving health intervention, equivalent to the EMIC for a given age k, and *HALY (k)* and *C (k)* are the expected future HALYs and health system costs for the person aged k years, respectively.

The components of the analysis, formula derivation, model, and assumptions are outlined below.

### Key components

The three main components in the analysis are health system costs, health gains, and the cost-effectiveness threshold.

#### Health system costs

- The one-off or direct cost of the life-saving health intervention itself is *A*.
- The estimated future health costs of the survivor aged k years after the life-saving intervention is *C(k)*, assuming he/she then adopts the same morbidity and mortality as the average citizen of the same age and sex and thus incurs the same future health system costs. This approach of considering future health system costs in survivors is probably the most appropriate one for modern health economic analyses [26]. The cost of publicly funded health services by age was from the New Zealand Ministry of Health database, called “HealthTracker” [14]. HealthTracker is a collection of linked administrative datasets of publicly funded health system events (including hospitalizations, mortality, cancer registrations, mental health and addiction service use, pharmaceutical and laboratory claims, primary health care enrollment, and outpatient/emergency department visits) for the entire New Zealand population with unit costs attached. But due to gaps in HealthTracker data the costs were scaled up across all age groups by 1.2. In addition, in the last year of life costs were multiplied by 1.1, 1.2, and 1.3 for the 65–74, 75–84, and 85+ age groups, respectively, to capture the estimated missing data of funding residential “disability support services” care funded by the government (Vote:Health) but not yet captured in available data.
- The total intervention cost for an individual aged k years is then the one-off direct cost of the intervention itself plus expected future health spending: *A + C(k)*.

#### Health gains

- The estimated future health gains of the survivor aged k years after the life-saving intervention is HALY (k). Health gain is captured in health-adjusted life-years (HALYs), assuming he/she then adopts the same morbidity and mortality as the average citizen of the same age and sex and gains the same future HALYs. The discounted future HALYs gained is the same as the discounted health-adjusted life expectancy (HALE) in this context. Incorporated within the HALY calculation is population background morbidity, in prevalent years of life lived in disability (pYLDs). These pYLDs were sourced from the New Zealand Burden of Disease Study [13] and divided by the number of people of the given sex and age to give an expected or average morbidity per person.

#### Cost-effectiveness threshold (CET)

- For the CET, and in the absence of any official New Zealand threshold, we used the rule-of-thumb approach proposed by the World Health Organization (WHO) as the departure point: those interventions with a cost-effectiveness ratio less than the country’s GDP per capita can be considered very cost-effective [27]. For this analysis, that equates to a conservative cost-effectiveness threshold of NZ$45,000 per HALY gained (for year 2011; $US30,000 adjusted for OECD purchasing power parity [28]). Sensitivity analyses using different CETs (NZ$20,000 and NZ$100,000) were undertaken.

### Formula derivation

Our basic premise is that in order for the maximum amount invested up front to be cost-effective, the total intervention cost per discounted HALY (otherwise known as the incremental cost-effectiveness ratio or ICER) has to be less than or equal to the CET.

1 $$ ICER=\frac{Total\  intervention\  cost- Comparator\  Cost}{Intervention\  HALY- Comparator\  HALY}\le CET $$

Assume there is a hypothetical intervention that prevents a death of a person aged *k* years with remaining lifetime *T* years.

The expected future HALYs for the person aged k years [*HALY (k)*] can be calculated as follows:

$$ HALY(k)={\displaystyle \sum_{i=k}^{\infty }}\frac{\left[1- pYLD(i)\right]}{{\left(1+r\right)}^{i-k+1}}*I\left\{T\ge i\right\} $$

Where pYLD is prevalent years of life lived in disability, a population background morbidity measure expressed as the average per person of a given sex and age. The discount rate is r.

Similarly, the expected future health costs for the person aged k years [C(k)] can be calculated as follows:

$$ C(k)={\displaystyle \sum_{i=k}^{\infty }}\frac{c_i}{{\left(1+r\right)}^{i-k+1}}*I\left\{T\ge i\right\} $$

$$ I\left\{T\ge i\right\}=\left\{\begin{array}{c}\hfill 1,\kern2.75em T\ge i\hfill \\ {}\hfill 0,\kern2.25em T<i\hfill \end{array}\right. $$

*C*_*i*_ is the average annual health care cost of a New Zealander aged *i* derived from the New Zealand Ministry of Health’s HealthTracker data [29].

As stated earlier:

$$ Total\  Intervention\  Cost=A+C(k) $$

$$ Intervention\  HALE= HALY(k) $$

On the other hand:

$$ Comparator\  Cost=0 $$

$$ Comparator\  HALE=0 $$

Therefore, the inequality (1) can be written as:

$$ CET\ge \frac{A+C(k)-0}{HALY(k)-0} $$

From here it immediately follows that EMIC is equal to *A (max)*, solved from the equation below:

$$ A(max)=\left(CET \times HALY(k)\right) - C(k) $$

### Model

To calculate HALYs and costs, we modeled the New Zealand population in 2011 with a simple, two-state Markov model (“Alive”-“Dead”), in TreeAge Pro 2012. The Markov model with an annual cycle followed the cohort (in fact an individual with given age) up to 105 years old. We used New Zealand population mortality rates from Statistics New Zealand [30] to calculate the probability of death. An individual with age i generates HALYs equal to 1-pYLD (i) and generates costs c (i) for the health system. Discounted 1-pYLD (i) and c (i) were assigned as health and cost “rewards” in the “Alive” state. Hence the model returned HALYs and costs for each modeled cohort. A 3 % discount rate was applied to all future health system cost and health gains. Sensitivity analyses of 0 % and 6 % were also conducted.

### Assumptions

For the New Zealand-specific and ‘ideal’ EMIC results that follow, we assume a hypothetical situation where the risk of death is 100 % (i.e., the individual will die without the intervention), this risk does not vary by age, and the risk persists for a duration of one year. The life-saving intervention is also assumed to be 100 % effective (i.e., it eliminates the risk of death completely). We applied a 2 % annual reduction to New Zealand mortality rates until 2026 and 1 % after that, to reflect improving life expectancy over time (these patterns reflect long-run mortality trends in the New Zealand context [31]). The online EMIC calculator (see below), however, allows the user to vary the anticipated decline in background mortality, risk of death over time, and other variables such as the risk of death without prevention or treatment interventions.

### Online calculator

The results that follow in the next section are specific to New Zealand. In principle, this approach can be applied to any other country as long as there are country-specific data on age and sex-specific mortality, morbidity (or combined as HALEs), and health system costs. Building on this idea, we have used available WHO data on HALE and health system expenditure by country [32, 33] to build an international EMIC calculator, available online at http://www.otago.ac.nz/wellington/research/bode3/otago078632.html.

The international EMIC calculator potentially allows researchers and policymakers to derive their own country-specific EMICs by selecting their country from a drop-down list, specifying the risk of death in the disease being considered as well as the effectiveness of the life-saving intervention, and selecting their preferred cost-effectiveness threshold. The calculator is relatively simplistic and users should be aware of several critical assumptions, outlined in full in the Additional file 1 and online user guide.

## Results

**Fig. 1:**
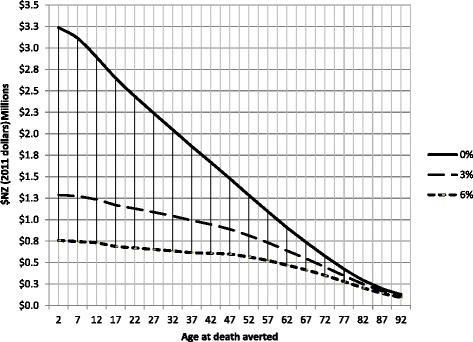
Estimated maximum intervention cost to save a life, at NZ$45,000 cost-effectiveness threshold, for three different discount rates

The EMIC to save a life for the ideal intervention (100 % fatal without intervention, 100 % effective intervention, no ongoing morbidity, mortality risk, or cost increase following intervention) varied markedly by age. Using a 3 % discount rate, the EMIC was NZ$1.3 million to save the life of a child, $0.8 million to save the life of a 50-year-old, and $0.235 million to save the life of an 80-year-old (Fig. 1, Table 1).

**Table 1:** Life expectancy, health-adjusted life expectancy (HALE), health system costs, and estimated maximum intervention cost (EMIC) at different discount rates

Age group	Life expectancy (years)	HALE (years)			Future (public) health system costs per individual^a^ per year (in thousands NZ$)			EMIC at a cost-effectiveness threshold of NZ$45,000 per HALY (in thousands NZ$)		
Discount rate:	0 %	0 %	3 %	6 %	0 %	3 %	6 %	0 %	3 %	6 %
0-4	88.2	75.8	28.8	16.4	$209	$42	$17	$3,236	$1,288	$757
5-9	82.8	70.7	28.0	16.2	$203	$43	$17	$3,011	$1,274	$746
10-14	77.4	65.5	27.2	15.9	$198	$47	$19	$2,788	$1,237	$734
15-19	71.9	60.4	26.2	15.5	$193	$51	$21	$2,544	$1,172	$695
20-24	66.6	55.6	25.4	15.3	$187	$54	$23	$2,335	$1,133	$682
25-29	61.4	50.9	24.4	15.0	$181	$57	$26	$2,134	$1,093	$672
30-34	56.2	46.2	23.3	14.6	$174	$61	$28	$1,936	$1,050	$662
35-39	51.0	41.5	22.1	14.2	$168	$64	$31	$1,733	$994	$640
40-44	45.8	36.9	20.8	13.7	$161	$68	$35	$1,538	$936	$618
45-49	40.6	32.4	19.2	13.1	$154	$73	$40	$1,341	$865	$584
50-54	35.7	28.1	17.6	12.3	$147	$76	$45	$1,153	$789	$546
55-59	30.8	23.8	15.7	11.4	$139	$79	$51	$972	$707	$500
60-64	26.1	19.7	13.7	10.2	$129	$81	$55	$795	$612	$441
65-69	21.6	15.9	11.6	9.0	$117	$80	$58	$637	$521	$384
70-74	17.2	12.4	9.5	7.6	$101	$74	$57	$490	$424	$320
75-79	13.0	9.1	7.4	6.1	$82	$65	$53	$357	$327	$252
80-84	9.3	6.3	5.4	4.6	$64	$53	$46	$245	$235	$186
85-89	6.3	4.2	3.7	3.3	$46	$40	$36	$162	$161	$132
90-94	4.1	2.7	2.5	2.3	$31	$28	$26	$106	$108	$92

^a^ Calculated at the midpoint of each age group, i.e., 2, 7, …, 92 years

Table 1 shows HALEs, future health system (publicly funded) costs, and corresponding EMICs by age. For example, for a 15 to 19-year-old male, at a CET of NZ$45,000 per HALY gained and a 3 % discount rate, expected HALE is 26.2 years and expected future health system (discounted) cost is $51,000. Hence, EMIC is equal to (26.2 × $45,000) - $51,000 = $1,128,000. Figure 1 shows EMICs by age for different discount rates in addition to the baseline rate of 3 % (i.e., 0 % and 6 %).

Sensitivity analyses show that the choice of the cost-effectiveness threshold (from NZ$30,000 to NZ$60,000 per HALY) has a large impact on the EMIC (Fig. 1, Table 2). This is especially the case for children. For example, at a discount rate of 3 %, the EMIC for a child’s life increases to $1.68 million (30 %) for a CET of $60,000 and decreases to $0.82 million (36 %) for a threshold of $30,000 (Table 2).

**Table 2:** Estimated maximum intervention cost (EMIC in millions NZ$) at different levels of cost-effectiveness threshold that reflect willingness-to-pay to save a HALY (at the 3 % discount rate)

Age group	Cost-effectiveness threshold for gaining a HALY								
	$20,000	$30,000	$40,000	$50,000	$60,000	$70,000	$80,000	$90,000	$100,000
0-4	0.53	0.82	1.11	1.40	1.68	1.97	2.26	2.55	2.84
5-9	0.51	0.79	1.07	1.35	1.63	1.92	2.20	2.48	2.76
10-14	0.49	0.76	1.03	1.30	1.57	1.85	2.12	2.39	2.66
15-19	0.46	0.73	0.99	1.25	1.51	1.78	2.04	2.30	2.56
20-24	0.44	0.69	0.95	1.20	1.45	1.71	1.96	2.21	2.47
25-29	0.41	0.66	0.90	1.14	1.39	1.63	1.88	2.12	2.37
30-34	0.39	0.62	0.85	1.09	1.32	1.55	1.79	2.02	2.25
35-39	0.37	0.59	0.81	1.03	1.25	1.47	1.70	1.92	2.14
40-44	0.36	0.57	0.78	0.98	1.19	1.40	1.61	1.81	2.02
45-49	0.35	0.55	0.74	0.93	1.12	1.32	1.51	1.70	1.89
50-54	0.32	0.50	0.67	0.85	1.02	1.20	1.37	1.55	1.73
55-59	0.28	0.44	0.60	0.75	0.91	1.07	1.23	1.38	1.54
60-64	0.24	0.38	0.52	0.65	0.79	0.93	1.07	1.20	1.34
65-69	0.20	0.32	0.43	0.55	0.67	0.78	0.90	1.02	1.13
70-74	0.16	0.26	0.35	0.44	0.54	0.63	0.73	0.82	0.92
75-79	0.12	0.19	0.27	0.34	0.41	0.49	0.56	0.63	0.71
80-84	0.08	0.13	0.19	0.24	0.29	0.35	0.40	0.45	0.51
85-89	0.04	0.08	0.12	0.16	0.19	0.23	0.27	0.30	0.34
90-94	0.02	0.05	0.07	0.10	0.12	0.15	0.17	0.20	0.22

## Discussion

### Main findings and interpretation

This paper presents one possible approach to estimating the maximum amount that a society could invest in a life-saving health intervention, at different ages, while remaining cost-effective from a health system perspective (by a specified threshold). Our EMIC estimates show marked variation by age: NZ$1.3 million (US$880,000) to save the life of a child, $0.8 million (US$540,000) to save the life of a 50-year-old, and $0.235 million (US$158,000) to save the life of an 80-year-old, for the scenario where death was inevitable and the intervention 100 % effective with no sequelae. The variation by age is not surprising given the central role that remaining life expectancy plays within a metric like health-adjusted life expectancy.

Although this case study used New Zealand data, this approach could potentially be employed in any other country as long as data on age and sex-specific mortality, morbidity (or combined as HALEs), and health system expenditure are available. HALE data are now readily available for many countries, for example from the recent Global Burden of Disease 2010 study [34]. The expected stream of future health spending is less readily available, although this is likely to change with the increasing linkage of health and costing datasets – and “like” country data can be used as a substitute. Using available WHO data, we have extended this analysis by building an international EMIC calculator that allows the user to apply this approach to specific countries (albeit under several critical assumptions). Results for other countries should, however, be used cautiously with due regard for the various limitations with EMIC calculations (as detailed further below and in the Additional file 1).

### How might EMIC results be used?

Policymakers often need to make decisions about which new health interventions to adopt or reject, and often quickly. These decisions are and should be based on multiple criteria, of which cost-effectiveness is just one (as detailed in the Introduction). But it is envisioned that calculating an EMIC for a possible intervention might be potentially useful as a rapid screening process. Obviously cost-effective interventions should be implemented and cost-ineffective interventions pursued no further, and borderline interventions should subjected to more thorough cost-effectiveness analyses. Moreover, EMICs might be useful in planning research, as researchers can determine whether the treatment or preventive intervention they are working on is likely to be less than the EMIC for a given society. For example, using these New Zealand results, if a life-saving intervention appears likely to cost many times more than NZ$1.3 million per life saved, then it is unlikely to be found cost-effective upon further, more detailed research in this setting for any age group (using a threshold of NZ$45,000 per HALY). Along the same lines, if a potential intervention appears to be likely to cost many times more than NZ$400,000 per life saved, further research may be unlikely to find it cost-effective in the 70-plus age group.

It is plausible that calculator-produced EMIC results could help with decision-making in a public health emergency, e.g., purchasing a new vaccine in the face of a pandemic emergency. But ideally documents such as pandemic plans should have analyses that are worked through in advance and cover a wide range of contingencies.

### Strengths and limitations of this study?

This study was able to use detailed epidemiological and health system costing data for one country. The EMIC values produced are age-specific and can reflect policymaker preferences around such aspects as discount rates, CET values, and future mortality trends. Furthermore, with the international EMIC calculator, EMIC estimates can be “customized” using country-specific mortality data, morbidity data, and health system costs, as well as permitting different CET values. Nevertheless, we recognize that this EMIC approach builds on the commonly used metrics of health economics (HALE, QALYs, and DALYs) which value future life expectancy and hence favor interventions aimed at younger ages. As such, a society that rejected any such weighting based on the magnitude of (discounted) remaining expected life expectancy should not use this type of approach. Nevertheless, for those societies that already use such metrics as QALYs and DALYs in health policymaking and which have explicit or implicit thresholds for determining what interventions are “cost-effective,” there is a logical coherence with using the EMIC approach as outlined here.

In our initial analyses, we used the CET approach from WHO-CHOICE (i.e., using a country’s GDP per capita for a HALY gained). Such an approach has been appropriately criticized (e.g., by Revill et al. [35], Newall et al. [36], and Marseille et al. [37]), and a particular weakness is that it is not linked to the shadow price of a health system’s budget constraint. But in the absence of viable alternative options, we used this approach as it still provides some indicator (albeit fairly crude) of the size of the resources that a country can draw upon to invest in health interventions. Furthermore, users are able to alter the CET as they see fit in the online calculator.

A further limitation of the EMIC approach is that it does not take into account any side effects of the intervention or any permanent morbidity sequelae or altered mortality risk among people whose life was saved. Such conditions will reduce the HALYs gained and therefore also reduce the amount that health system policymakers would be willing to spend on the intervention for a given CET. As stated earlier, it is not so applicable to chronic diseases that reduce life expectancy, but rather is best when considering relatively acute, “short and sharp” diseases or injuries, where the intervention that saves the lives of individuals then typically returns them to expected health.

### Potential further research and country-specific adaptation

Given the limitations outlined above, it would be ideal if different societies conducted research on whether or not their populations value life differently across the life course or if they philosophically object to any such differentials. This could be explored with surveys of values or potentially with citizen juries [38]. Similar means can be used to determine the appropriate discount rate to use for country-specific health system decision-making.

In terms of the use of the EMIC results produced here and from the online calculator, it would be useful to research the utility of these for busy policymakers. Does this information allow for the use of limited health economic modeling research resources to be better targeted? Or will policymakers find the residual uncertainty too large to make any such early calls about not researching the cost-effectiveness of potential interventions further?

Finally, in the New Zealand setting, there is a need for ongoing refinements in health system costs as outlined in a 2014 article [14] and subsequent work on health costs (submitted for publication in 2015). Moreover, for international applications of the EMIC, country-specific cost data would be ideal, or at least some knowledge as to whether New Zealand cost data can be appropriately scaled up or down.

In conclusion, we present a possible approach to estimating maximal cost-effective investment in life-saving health interventions, under various assumptions and scenarios. Our online calculator allows this approach to be applied for a range of user-specified assumptions and inputs and in other countries. Despite the various limitations with this work, busy policymakers could use these estimates as a rapid screening tool. This could determine if more detailed cost-effectiveness analyses of potential life-saving interventions might be worthwhile or which proposed interventions are very unlikely to benefit from such additional research.

## Additional file

Additional file 1:
**Outline and assumptions of the International EMIC calculator.**
