# Supplementary material for: How much might a society spend on life-saving interventions at different ages while remaining cost-effective? A case study in a country with detailed data
Source: Popul Health Metr. 2015 Jul 8;13:15. doi: 10.1186/s12963-015-0052-2 (PMC4493819; doi:10.1186/s12963-015-0052-2)
Supplement: Additional file 1: — Outline and assumptions of the International EMIC calculator. [file 12963_2015_52_MOESM1_ESM.docx]

**Additional file 1: Outline and assumptions of the International EMIC calculator**

Giorgi Kvizhinadze, Department of Public Health,

University of Otago, Wellington, New Zealand,

Email: [giorgi.kvizhinadze@otago.ac.nz](mailto:giorgi.kvizhinadze@otago.ac.nz)

This Appendix describes how the “Estimated Maximum Intervention Cost” (EMIC) approach (applied to New Zealand in the main manuscript) can be applied to other countries, via our online International EMIC calculator available online (<http://www.otago.ac.nz/wellington/research/bode3/otago078632.html>).

The calculator works by using available WHO data on health-adjusted life expectancy (HALE) and total health system expenditure by country.(1, 2) We start with New Zealand (NZ) mortality rates, morbidity rates, and health system costs-and scale these up or down for other countries as necessary. In other words, we scale NZ mortality rates to achieve the life expectancy of other countries, we scale NZ morbidity rates to achieve the HALE of other countries, and we scale costs to match the percentage of GDP spent on health in other countries. Calculations for other countries are undertaken using NZ dollars, and then converted to either US dollars or local currency using exchange rates.(3) The scaling process is outlined in more detail below:

Step 1: **Scale up NZ public health system costs up to the approximate NZ total health expenditure**

In the main manuscript, calculations are done from only the public health system perspective. The calculator however uses *total* health expenditure. Approximately 82.3% of total expenditure on health in NZ is funded publicly, and the remainder funded privately. Hence, to estimate total health system expenditure in NZ we inflate the cost of publicly-funded health services by 1/0.823.(4) This value (NZ total health system costs for the average citizen) serves as a reference point for the total health expenditure of other countries, and is also used to estimate the maximum amount that society can invest to eliminate a specified risk of death.

Step 2: **Scaling NZ total health system cost to approximate other countries’ total health system cost**

Using World Bank data, total health expenditure as a percentage of GDP and GDP per capita in US dollars by country were obtained.(2) Total health expenditure was first estimated. Then, the ratio between the country-specific value and the NZ value was used as a scalar, and applied to NZ average citizen costs.

## Step 3: Scaling NZ mortality and morbidity to approximate other countries’ mortality and morbidity

Life expectancy (LE) and health-adjusted life expectancy (HALE) were obtained from the WHO website(1) and were used to scale country-specific mortality rates and morbidity rates (via prevalent years of life lived in disability or pYLDs). The scalars for mortality rates and pYLDs were solved from two Markov models generating observed differences between LE and HALE of country of interest and NZ.

**Critical assumptions and limitations**

The International EMIC calculator has several critical assumptions, namely:

1. The distribution of mortality rates, morbidity rates, and health system costs by age in other countries is the same as in NZ.
2. The age structure of other countries is similar to NZ (relevant to costs and using percentage GDP on health as a scalar).

Therefore the calculator may be less suited to countries with very different age structures, different spending patterns by age (NZ spends relatively large amounts of health system funds on older citizens), or with different morbidity patterns by age (for example developing countries may have much higher morbidity at younger ages from diarrheal or respiratory diseases compared to NZ). In general this calculator is likely to be more accurate for developed or high-income countries.

The other limitations of the EMIC approach still apply to the calculator, namely:

- That it is suited towards assessing life-saving interventions in relatively acute ‘short and sharp’ diseases or injuries, where the intervention saves their life and then returns them to expected health, having the same morbidity and mortality as the average citizen of their age and sex.
- That we only consider health system costs and do not include economic productivity, etc.
- That the calculator assumes the life-saving intervention is 100% effective, i.e. it completely eliminates the risk of death.

**References**

1. World Health Organization. Global Health Observatory Data Repository: Life Expectancy Data by Country. World Health Organization; 2014 [cited 2014 September 15]; Available from: <http://apps.who.int/gho/data/node.main.688?lang=en>

2. World Health Organization. Global Health Observatory Data Repository: Health expenditure per capita-Data by country. World Health Organization.; 2014 [cited 2014 September 15]; Available from: <http://apps.who.int/gho/data/node.main.78?lang=en>

3. The World Bank. Official exchange rate (LCU per US$, period average). The World Bank; 2014 [cited 2014 September 15]; Available from: <http://data.worldbank.org/indicator/PA.NUS.FCRF>.

4. Blakely T, Foster R, Wilson N. Burden of Disease Epidemiology, Equity and Cost-Effectiveness (BODE3) Study Protocol. Wellington: Department of Public Health, University of Otago, 2012.
